# Hyaluronic acid gel application versus ovarian suspension for prevention of ovarian adhesions during laparoscopic surgery on endometrioma: a double-blind randomized clinical trial

**DOI:** 10.1186/s12905-022-01607-2

**Published:** 2022-02-11

**Authors:** Shahla Chaichian, Seyed Reza Saadat Mostafavi, Abolfazl Mehdizadehkashi, Zahra Najmi, Kobra Tahermanesh, Mahin Ahmadi Pishkuhi, Fatemeh Jesmi, Bahram Moazzami

**Affiliations:** 1 Pars Advanced and Minimally Invasive Medical Manners Research Center, Pars Hospital, Iran University of Medical Sciences, Tehran, Iran; 2 Department of Radiology, Iran University of Medical Sciences, Tehran, Iran; 3 Endometriosis Research Center, Iran University of Medical Sciences, Tehran, Iran; 4 Zanjan University of Medical Sciences, Zanjan, Iran

**Keywords:** Adhesions, Hyaluronic acid, Laparoscopy, Endometriosis, Ovarian suspension

## Abstract

**Background:**

This study aimed to compare the effect of ovarian suspension and hyaluronic acid gel to prevent re-adhesions after laparoscopic endometrioma surgery.

**Methods:**

This randomized clinical trial was conducted at Rasoul-e-Akram and Pars Hospitals, Tehran, Iran, 2016-18. Fifty patients with bilateral endometrioma and pelvic adhesions, the candidates of laparoscopic surgery, were included. In each patient, at the end of ovarian cystectomy and adhesiolysis, one of the ovaries was randomly sutured to the abdominal wall, and the HYAcorp Endogel covered the other; the adhesion rate was compared between the groups by ultrasonography, three-month after surgery.

**Results:**

Mean age of patients was 32.6 years. Presurgical variables were similar between right and left ovaries and the study groups (P > 0.05). Postsurgical ultrasonography showed that ovarian soft markers, including < 1/3 ovarian adhesions (minimal adhesions) in 80.5% of ovaries of the Endogel group and 35.5% of ***the*** ovarian suspension group (P < 0.001) with higher ovarian mobility in the Endogel group (65% vs. 22%) (P = 0.001). In addition, site-specific tenderness and ovarian fading margin were lower in the Endogel group (P < 0.001).

*Trial registration* Clinical trial registry number: IRCT2015081723666N1, 12.19.2015, Date of registration: 01/02/2016; https://en.irct.ir/trial/20174?revision=20174. Date and number of IRB: 2015, I.R.IUMS.REC.1394.24703.

**Conclusion:**

Hyaluronic acid gel can be more effective than ovarian suspension in preventing ovarian adhesions after laparoscopic treatment of endometriosis.

## Introduction

Adhesions are considered an important etiology of pain, infertility, bowel and ureteral obstructions in patients with endometriosis [1] and are supposed to be formed by inflammation, reduced apoptosis, and increased angiogenesis and neurogenesis in endometriotic tissues [2], significantly intensified at higher stages of endometriosis [3]. As adhesions can make the surgical procedure more complicated and time-consuming and cause several problems, such as the continuation of pain and infertility, it is necessary to reduce the risk of adhesion in each surgical procedure [4].

Although numerous surgical techniques, such as ovarian suspension, traditionally used to separate the ovaries from the pelvis [5–7], or other preventive methods [8], by using normal saline, heparinized lactated ringer solution, corticosteroids, and peritoneal lavage by Dextran 32% [9], polytetrafluoroethylene (Gore-Tex) and oxidized regenerated cellulose (Interceed), chemically modified sodium hyaluronic acid/carboxymethylcellulose (Seprafilm) [10] have been approved as an efficient method for the adhesion prevention, none of them could completely prevent adhesion recurrence after laparoscopic surgery for endometriosis.

Hyaluronic acid gel, known as hyalobarrier gel (used under different brands), is suggested to be used alone or in combination with carboxymethylcellulose, membrane to prevent adhesions [11, 12]. Furthermore, in the present study, we aimed to compare the effect of hyalobarrier gel and ovarian suspension during laparoscopic cystectomy for treatment of bilateral endometrioma on postoperative pelvic adhesions. To reduce the confounding effect of different immunological and inflammatory responses of the endometriotic patients, we randomized the ovaries instead of randomizing patients, as previously used in other bilateral organs [13]. We evaluated the postoperative pelvic adhesions by ultrasound examination as an accurate diagnostic tool for assessing pelvic adhesions in endometriotic patients [14, 15].

## Methods

### Study design

In the present randomized clinical trial (RCT), patients with severe endometriosis (stages 3 or 4; according to rASRM staging system for endometriosis and bilateral endometrioma), who referred to Rasoul-e-Akram and Pars Hospitals, Tehran, Iran, for laparoscopic surgery during 2016 to 2018 were included into the study. To eliminate or reduce the effect of genetic, epigenetic, and immunologic factors, we decided to allocate the ovaries rather than the patients, so 100 ovaries were recruited as the case/control groups of the study. For the allocation of ovaries, a simple randomization technique by application of quadruple blocks was used. We used concealed envelopes opened by a technician that informed the surgeon during laparoscopy for the concealment. For blinding patients, the sutures for ovarian suspension and wound repair on the opposite abdominal wound were done by 3-0 Vicryl and cut simultaneously on the 3rd day of the surgery. The sonologist wasn't aware of the surgical site.

In this study, the purpose of ovarian suspension was not clearance of the surgical field. Instead, we aimed to make the ovary far away from the pelvis during the first three days of surgery to prevent scar tissue formation around it and prevent the anti-adhesive effect of endogel applied around the opposite ovary. We could not consider internal suspension because we have to release the suspended ovary before ending the surgery.

According to Dhanawat's study [16], three days of ovarian suspension is an appropriate length of time for preventing adhesion. At the end of 72 h, the suspended ovary should be returned to the pelvic cavity by releasing the suspension suture. This is especially important for endometriosis patients who may need ART, that oocyte retravel is crucial and necessitates the appropriate pelvic positioning of the ovary.

The Ethics Committee of the Iran University of Medical Sciences approved the study protocol.

Ethics code: I.R.IUMS.REC.1394.24703) and registered on the Iranian RCT website (IRCT2015081723666N1).

### Sample size

The study sample size was calculated at 50 ovaries in each group, using the formula for binary dependent variables, considering an alpha error of 0.05, study power of 80%, and minimum clinically significant difference in the prevalence of postoperative ovarian adhesion between two study groups at 50% reduction. According to Hoo et al. [6], the prevalence of postoperative ovarian adhesions as ***the*** primary outcome in the ovarian suspension group was 38.5% and in the unsuspended group was 51.9%.

The study's inclusion criteria consisted of women of reproductive age with clinical and ultrasonography diagnosis of bilateral endometrioma and pain score > 7, who were candidate for laparoscopic surgery and have signed the written informed consent form for the study. Every patient who did not sign the consent form was not included in the study. The exclusion criteria encompassed patients diagnosed with unilateral endometrioma or another cyst type (rather than endometrioma) during surgery. Patients who did not refer for a follow-up examination and or rejected to continue were excluded from the study.

The gynecologist diagnosed endometriosis based on clinical and imaging criteria. Diagnosis of bilateral endometrioma and presence of adhesions were confirmed by the same sonographer. During gynecological examination, patients' pain severity was evaluated by visual analog scale (VAS) and marked by patient herself on a 10-point Likert scale; patients with a score ≥ 7, who were irresponsive to medical treatment, were invited to participate to the study. Patients with unilateral endometrioma or deep endometriosis (D.E.) were not included in the study. Before enrolling patients into the study, the researcher explained them the study objectives to the eligible patients and asked them to read and sign the written informed consent. All patients were referred to our infertility clinic for standard recommendations by fertility experts and possible fertility preservation.

### Data collection

**Fig. 1:**
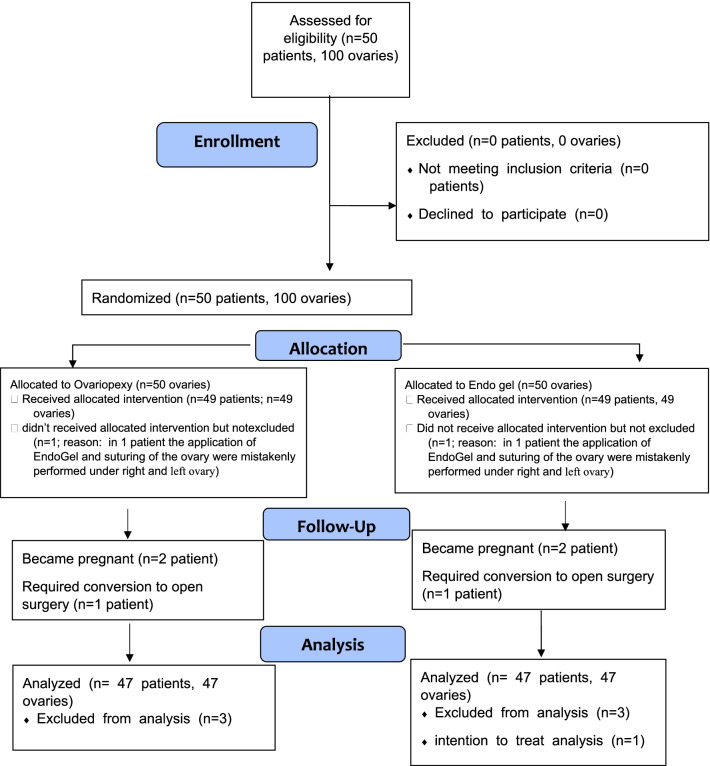
CONSORT 2010 flow diagram of patients’ enrollment into the study

Patients' demographics, including age, marital status, and body mass index (BMI), were recorded from the hospital's medical records. Hormonal medications were discontinued three months before laparoscopy (washout period). The ovaries were allocated into two groups of ovarian suspension and hyaluronic gel application using quadruple block randomization, prepared by a statistician, by simple randomization method using Excel software without duplicates. The CONSORT 2010 flow diagram (Fig. 1) shows the process of sampling and ovarian allocation and utilization of intention to treat policy for analysis. In the operating room, the responsible technician was asked to open the result of the randomized block to declare the side of ovarian suspension and hyaluronic gel application for performing the allocation. Patient and sonographer were unaware of the group allocation, and the analyst also analyzed the data with codes instead of patients' names.

### Surgical techniques

**Fig. 2:**
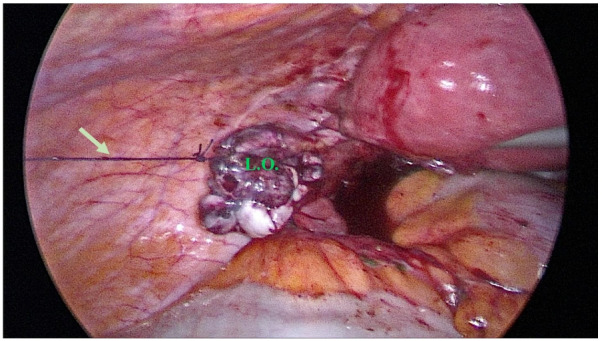
A panoramic view of pelvic cavity after suspension of the left ovary to abdominal wall, according to block randomization

**Fig. 3:**
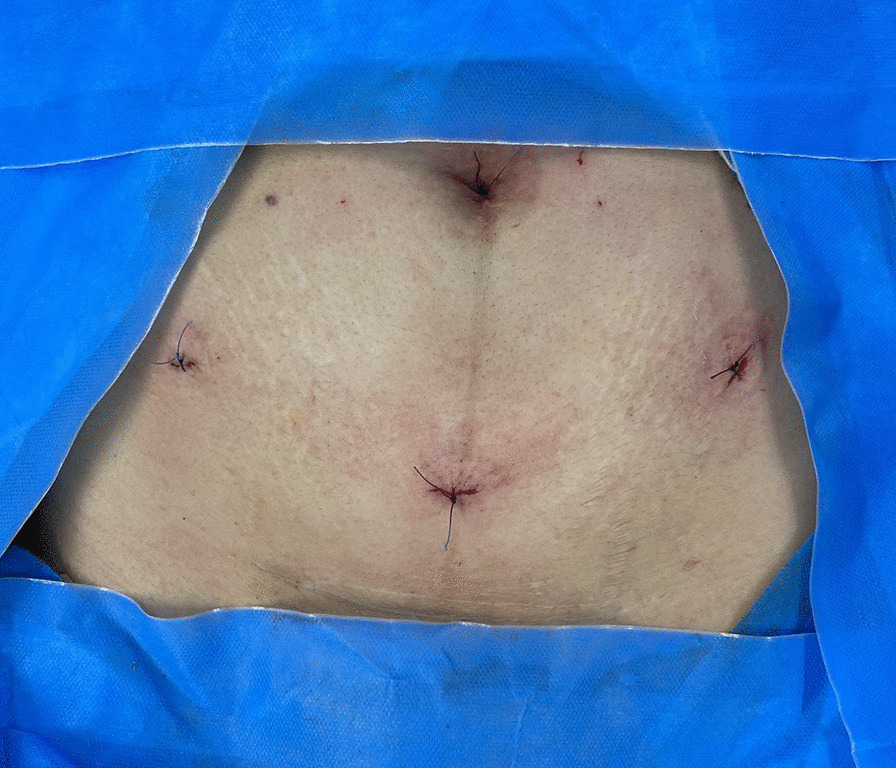
The abdomen’s view at the end of procedure. Note that the suspension thread (3-0 Vicryl) exited from the right 5-mm trocar incision and the wound repaired by the same size suture. Then right 5-mm incision was repaired similarly by 3-0 Vicryl (We ordinary repair these incisions by 4-0 Vicryl sutures) to blind the patient from treatment modalities

All patients underwent laparoscopic surgery by the same surgical team. After direct umbilical trocarization by 11-mm trocar, carbon dioxide (CO_2_) insufflation was performed. Then two 5.5-mm side trocars and one 11-mm suprapubic trocar were inserted. Abdominal and pelvic cavity exploration was done by a zero-degree optic, and bilateral endometriomas were confirmed. The ovarian adhesions to the uterus, contralateral ovary, bowel, and abdominal wall were released. Each ovary was opened by scissor, and the cyst wall was separated from the ovarian tissue by gentle tractions and counter tractions, as much as possible, and by opening the endometrioma, its content was aspirated, and the ovaries were repaired using 3-0 Vicryl by mattress suture, after careful hemostasis, preferably by sutures. Based on the randomization method, one ovary was sutured by 3-0 Vicryl to the abdominal wall (Fig. 2); the suture thread was brought out at the site of the relevant 5.5-mm trocar and then another 5.5-mm trocar site was sutured by 3-0Vicryl too, so the patient couldn't find out the ovarian suspension side by looking at the suture material (Fig. 3). Another ovary was covered by one sterile pre-filled HYAcorp Endogel (BioScience GmbH, Germany) container, injected all around the ovary via laparoscopic needle. On the third day, the suspended ovary and other abdominal sutures were released by cutting the sutures [16, 17]. Any patient who was diagnosed with unilateral endometrioma or any other cyst (rather than endometrioma) during surgery, patients who did not refer for a follow-up examination, and or rejected to continue the study were excluded from the study.

### Study outcomes

The study's primary outcome was three-months pelvic adhesions surveillance, evaluated by transvaginal ultrasound and comparison with presurgical indices. Revised ASRM classification and ultrasonography soft markers (ovarian mobility, site-specific tenderness [SST], and ovarian fading margin) of endometriosis were used to examine the incidence and severity of ovarian adhesions. The secondary outcomes were adhesions to the bladder, ovary, bowel, anterior and posterior peritoneum, pelvic and ovarian adhesions, and maximum ovarian diameter were determined by the sonographer and recorded in the study checklist before and three-months after surgery. Any patient who required conversion to laparotomy for any reason or became pregnant during the follow-up period was excluded from the study.

### Statistical analysis

The data were described using frequency (percentage) for categorical variables, mean ± standard deviation (S.D.) for numeric variables with a normal distribution, and median (interquartile range) for numeric variables without normal distribution, based on the results of the Kolmogorov Smirnov test. According to the results of this test, in case of rejection of the normal distribution of the data, Wilcoxon test was used to compare the numeric variables among the groups. Mc Nemar's was used to compare the percentage of interested outcomes among the study groups. The statistical software IBM SPSS Statistics for Windows version 21.0 (IBM Corp. 2012. Armonk, NY: IBM Corp) was used for the statistical analysis. P values of < 0.05 were considered statistically significant.

## Results

One hundred ovaries of 50 patients were included in the study. Two patients became pregnant during the three-months follow-up period, and one patient required laparotomy for bowel resection. So, six ovaries were excluded from the study. In one patient, the application of Endogel and suturing of the ovary were mistakenly performed on the right and left ovary. Still, we decided not to exclude these two ovaries and counted them as the intention to treat group. So, finally, data of 47 patients and 94 ovaries were analyzed (Fig. 1). The mean age of patients was 32.6 ± 4.12 years (minimum of 24 and maximum of 42 years), mean BMI was 23.79 ± 3.07 kg/m^2^, and 59.6% of participants were married. In each patient, both ovaries (94 ovaries) were evaluated and treated by either ovarian suspension or Endogel.

Table 1 indicates the preoperative characteristics of ovaries. As shown, there were no differences in the characteristics of the ovaries of the two arms of the study, including the maximum diameter of the ovary, ovarian margin adhesion, endometrioma, and ovarian mobility (P > 0.05). Moreover, the preoperative characteristics of ovaries, including ovarian margin adhesion, mobility, and fading margin, were not different among the study groups (P > 0.05; Table 1).

**Table 1:** Comparing the characteristics of right and left ovaries before surgery in the studies patients

	Right ovary	Left ovary	p-value	Ovarian suspension	Endo gel	p-value
*Maximum ovarian diameter, Number (percent)*						
< 3	0	1 (2.1%)	.189	–	–	–
3–6	12 (24.5%)	14 (29.7%)		–	–	–
> 6	35 (74.5%)	32 (68.2%)		–	–	–
*Ovarian adhesion*						
< 1/3	0	0	.304	0	0	.060
1/3–2/3	4 (8.5%)	13 (27.7%)		5 (10.6%)	12 (25.5%)	
> 2/3	43 (91.5%)	34 (72.3%)		42 (89.4%)	35 (74.5%)	
*Site specific tenderness*						
No	1 (2.1%)	2 (2.1%)	1.00	0	1 (2.1%)	1.00
Yes	46 (97.9%)	45 (95.7%)		47 (100%)	46 (97.9%)	
*Endometrioma*						
No	1 (2.1%)	0	Not computable	–	–	–
Yes	46 (97.9%)	47 (100%)		–	–	–
*Ovarian mobility*						
Yes	24 (51.1%)	13 (24.4%)	.096	21 (44.7%)	14 (29.8%)	.180
No	23 (48.9%)	34 (75.6%)		26 (55.3%)	31 (70.2%)	
*Ovarian fading margin*						
No	–	–	–	11 (23.4%)	7 (14.9%)	.661
< 1/3	–	–	–	19 (40.4%)	18 (38.3%)	
1/3–2/3	–	–	–	10 (21.3%)	12 (25.5%)	
> 2/3	–	–	–	7 (14.9%)	10 (21.3%)	

As indicated in Table 2, after laparoscopy, the Endogel group had a lower frequency of ovarian adhesion > 1/3 and site specific tenderness (SST) (both P < 0.001) and a higher frequency of positive ovarian mobility (P = 0.001) and fading ovarian margin < 1/3 (P < 0.001), compared to the ovarian suspension group.

**Table 2:** Comparing the post-intervention ovarian characteristics between study groups

	Ovarian suspension (47 ovaries)		Endo gel (47 ovaries)		p-value*
	Frequency	Percent (%)	Frequency	Percent (%)	
*Ovarian adhesion*					
< 1/3	17	38.5	37	80.5	< .001
1/3–2/3	23	53.8	7	14.6	
> 2/3	6	7.7	2	4.9	
*Site specific tenderness*					
No	18	38.3	35	74.4	< .001
Yes	29	61.7	12	25.6	
*Ovarian mobility*					
Yes	9	22.0	26	65.0	.001
No	32	78	14	35	
*Ovarian fading margin*					
No	11	23.4	10	21.3	< .001
< 1/3	13	27.6	31	66	
1/3–2/3	20	42.5	4	8.5	
> 2/3	3	6.5	2	4.2	

*P-values < .05 are considered significant, calculated based on the results of chi square test

Comparing the frequency of adhesions at different sites before and three-months after the surgery showed that the frequency of adhesion to the bladder, right or left ovary, large and small bowel, anterior and posterior peritoneum, right and left pelvic areas, as well as sliding signs did not significantly change after the intervention (P > 0.05, Table 3).

**Table 3:** Comparing the rate of adhesions before and after surgery in the studied population

	Before surgery		Three months after surgery		p-value*
	Frequency	Percent	Frequency	Percent	
*Adhesion to bladder*					
No	13	27.7	39	82.9	.462
Mild to moderate	27	57.4	3	6.4	
Severe	7	14.9	5	10.6	
*Adhesion to right ovary*					
No	0	0	17	38.6	.256
Mild to moderate	3	6.4	21	47.7	
Severe	44	93.6	6	13.6	
*Adhesion to left ovary*					
No	0	0	22	50	1.00
Mild to moderate	2	2.1	19	43.2	
Severe	45	47.9	3	6.8	
*Adhesion to colon*					
No	0	0	25	55.6	.520
Mild to moderate	2	2.1	19	42.2	
Severe	45	47.9	1	2.2	
*Adhesion to small intestine*					
No	31	66	47	100	–
Mild to moderate	12	25.5	0	0	
Severe	4	8.5	0	0	
*Adhesion to anterior peritoneum*					
No	9	19.1	47	100	–
Mild to moderate	31	66	0	0	
Severe	7	14.9	0	0	
*Adhesion to posterior peritoneum*					
No	0	0	2	4.4	–
Mild to moderate	1	2.1	43	95.6	
Severe	46	97.9	0	0	
*Adhesion to right pelvic area*					
Mild	5	10.6	15	32	1.00
Moderate	13	27.7	5	10.6	
Severe	29	61.7	0	0	
*Adhesion to left pelvic area*					
Mild	5	10.6	15	32	1.00
Moderate	13	27.7	5	10.6	
Severe	29	61.7	0	0	
*Sliding sign*					
No	30	63.8	30	63.8	1.00
Decreased	16	34	15	31.9	

*P-values < .05 are considered significant, calculated based on the results of chi square test

## Discussion

The ultrasonographic parameters (ovarian adhesion, mobility, fading margin, and SST) showed the superiority of the application of Endo gel on the ovaries compared to ovarian suspension within three months. These results are in agreement with previous studies, suggesting Endogel as an effective adhesion-preventing factor [11, 12] in gynecologic laparoscopic and hysteroscopic surgery [18]. However, they have not addressed ovarian adhesions solely and have considered various gynecological procedures. HYAcorp Endogel is shown to be superior to lactate ringer solution on preventing postoperative adhesions after laparoscopic ovarian drilling in patients with polycystic ovarian syndrome (PCOS) [19], which confirms the results of the present study. However, the type of disease, surgical procedure, and the control group were different. Confirming the current study results, a review of adhesion preventive techniques showed that H.A. alone or cross-linked with various agents such as nanoparticles are efficient easy-to-use gel, suggested to be used around the adnexal region or myomectomy site in gynecological diseases [20]. The mechanism of this efficacy is that this glycosaminoglycan, one of the components of the extracellular matrix, deposits around the surgical site and reduces the chance of adhesion formation with favorable biocompatibility and safety profile [21, 22]. On the contrary, comparing the effect of Hyalobarrier® with the control group (no intervention) in women with periadenexal adhesions at the time of laparoscopy showed the influence of Hyalobarrier® neither on adhesion and pregnancy rate two years after surgery, nor on follicular development (three months after surgery) [23]. This difference between the results of this study and ours could be due to the different H.A. brand use, as well as the fact that we have not compared the results of the two brands but with another intervention (ovarian suspension). As the severity of adhesion and infertility depends on genetic and epigenetic characteristics and immunologic and inflammatory responses of the individuals [24], we allocated the ovaries to control the effect of this confounding factor via using both allocation methods in every patient [6].

Ovarian suspension or oophoropexy, performed by different techniques, maintains the ovaries suspended far from the pelvic organs [25]. The choice of time for releasing the suspended ovaries from the abdominal wall was based on the study by Landi et al., which considered ovarian suspension release three days after surgery [17]. In a recent survey, Dhanawat and colleagues also confirmed the formation of fibrin deposits as early as 3 h after injury and suggested fibrinolysis formation three days after surgery [16]. In a previous study, unilateral or bilateral transient ovarian suspension of 336 ovaries showed a reduced risk of adhesion formation by distancing the ovaries from the pelvic cavity during wound healing in patients who underwent surgery for severe endometriosis [26]. All of the ultrasonographic criteria, including ovarian adhesion and soft markers, were better in the Endogel group, which could be due to more manipulation and foreign body (suture material) in the ovarian suspension group. The higher rate of ovarian fading margin in the Endogel group may be due to the intrinsic effect of non-absorbed Endogel around the ovary. Despite the significant reduction in severe ovarian adhesions on both sides, the comparison of postoperative values with the preoperative values was not statistically significant. Furthermore, neither of the techniques used in this study could influence the formation of adhesion in other sites, such as bladder, large and small bowel, anterior and posterior peritoneum, right or left pelvic areas.

The limitations of the present study included the nonrandomized inclusion of patients into the study, which decreases the generalizability of the results. Furthermore, we have considered the patients three-months follow-up results. In contrast, longer follow-ups can better indicate the efficacy of treatments and evaluate their effectiveness on long-term clinical outcomes, such as pregnancy rate. Besides, we recorded the ultrasonographic results for adhesion and did not include patients' clinical symptoms or folliculogram, while not all patients with adhesions are symptomatic.

## Conclusions

The present study results on patients with bilateral ovarian adhesions associated with severe endometriosis showed that HYAcorp Endogel could effectively reduce the risk of adhesion compared to ovarian suspension three months after surgery. Future studies can indicate the long-term outcome of using Endo gel, compared to other techniques, on the rate of adhesion, pregnancy, etc., and demonstrate the most effective and safe strategy for adhesion prevention.

## Data Availability

The datasets used and or analyzed during the current study are available from the corresponding author on reasonable request.
